# Polyglycolic acid sheet with fibrin glue potentiates the effect of a fibrin-based haemostat in cardiac surgery

**DOI:** 10.1186/1749-8090-9-121

**Published:** 2014-07-08

**Authors:** Hirofumi Kasahara, Ichiro Hayashi

**Affiliations:** 1 Department of Cardiovascular Surgery, National Hospital Organization Saitama Hospital, 2-1 Suwa, Wako 351-0102, Japan

**Keywords:** Polyglycolic acid sheet, TachoComb, Hemorrhage, Left ventricle

## Abstract

**Background:**

Hemorrhage from the left ventricle can be critical and sutureless repair using a fibrin-based haemostat (TachoComb) is one effective option. When active hemorrhage is not controlled by the haemostat, we have used a polyglycolic acid (PGA) sheet and fibrin glue in addition. Here we investigated whether the PGA sheet and fibrin glue combined with TachoComb had stronger adhesive properties than TachoComb alone in two experimental models.

**Methods:**

Experiment 1. An airtight circuit that included rabbit skin with holes covered by each type of sealant was gradually pressurized and the burst pressure was recorded automatically (n = 10). Experiment 2. A suture loop was attached to a porcine heart by each sealant, and the peel-off pressure was measured (n = 12).

**Results:**

The PGA sheet and fibrin glue combined with TachoComb showed significantly higher adhesive strength than TachoComb alone in both experiments (p < 0.05).

**Conclusions:**

Adding a PGA sheet and fibrin glue increased the adhesive strength of TachoComb in two experimental models, suggesting that this method might be effective for achieving haemostasis in difficult clinical situations.

## Background

Active hemorrhage from a ventricle is potentially catastrophic. Critical left ventricular (LV) wall injury can be caused by myocardial infarction, catheter intervention, cardiac surgery, or trauma. Recent changing trends in the population at risk have resulted in an increasing number of high-risk patients undergoing coronary intervention and cardiac surgery, leading to a potential increase in the risk of such complications. Sutureless repair methods have been reported for ventricular injury, especially using TachoComb (TC) or TachoSil, which are biodegradable collagen patches with a fibrinogen/thrombin-based coating. In severe cases, such as active bleeding from the left ventricle or LV free wall rupture, we have applied a polyglycolic acid (PGA) mesh sheet with fibrin glue in addition to TC in order to achieve stronger adhesion, and we have the clinical impression that this method is often effective even in dire situations.

Polyglycolic acid is gradually degraded by hydrolysis and slowly loses strength in vivo, and it is currently used to make absorbable surgical sutures. Polyglycolic acid mesh sheet is a soft nonwoven fabric known for its flexibility, elasticity, and low cost, which is available in the EU countries and Japan for clinical use as an absorbable reinforcing material. Since PGA itself is not adhesive, fibrin glue is usually combined with PGA sheets for surgical use. There have been many reports about the use of PGA sheets with fibrin glue for the treatment of lung injury or pulmonary artery damage in the field of thoracic surgery [1,2]. However, there have been no reports concerning PGA sheets as a haemostatic material for cardiac surgery to our knowledge.

## Methods

The polyglycolic acid sheet used in this study (Neoveil, Gunze, Kyoto, Japan) was a 0.15 mm thick biodegradable synthetic nonwoven fabric that is absorbed about 15 weeks after implantation. In order to evaluate the usefulness of combining a PGA sheet and fibrin glue (Bolheal, Kaketsuken, Kumamoto, Japan) with TC (Nycomed Pharma, Linz, Austria), we conducted the following 2 experiments to assess adhesive force.

**Figure 1:**
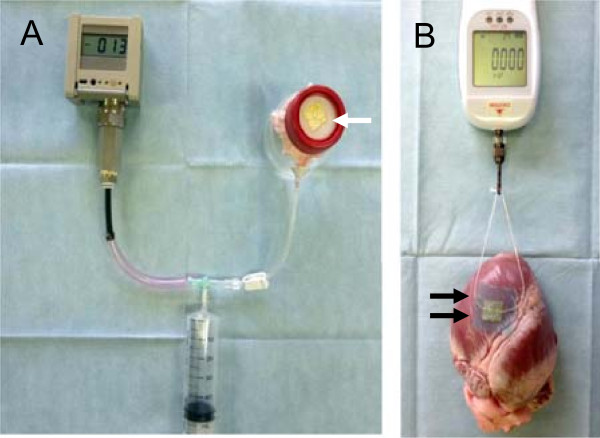
**Experimental design. A**: Experiment 1; An airtight circuit is formed from a pressure gauge, a plastic syringe, and a pressure-hardened plastic tank covered by rabbit skin with 5 holes. The holes are covered with TachoComb sheets (arrow). **B**: Experiment 2; A silk suture loop was placed on the pericardium and fixed with a TachoComb sheet plus a PGA sheet and fibrin glue (arrows). After stabilization, tension was gradually applied to the loop with a digital gauge.

Experiment 1: An airtight circuit was made with a digital pressure gauge (PG-208-102GH, Copal Electronics Co., Tokyo, Japan), a plastic syringe, and a pressure hardened plastic tank with an opening at the top [2] (Figure 1A). A sheet of skin from a Japanese white rabbit with 5 holes 2 mm diameter in a 10 mm square created by a template and a metal puncher was attached to the top of the tank. The holes in the skin sheet were covered by TC with (n = 5)/without a PGA sheet and fibrin glue (n = 5), as follows. Either a 15 mm square of TC was compressed manually over a hole with moist gauze for 3 minutes by a technician, or else a 30 mm square PGA sheet was placed over the center of the TC sheet, attached with 0.5 ml of fibrin glue, and stabilized for 3 minutes. Fibrin glue was applied by spraying from the nozzle of the spray applicator, which mixed the two components of the glue (fibrinogen and thrombin solutions). Then the pressure of the airtight circuit was gradually increased using a syringe pump at a rate of 2 mmHg per second approximately, and the maximum pressure at the rupture point (the onset of air leakage) was recorded automatically to assess the adhesive strength of each sealant. The rabbit skin sheet was exchanged for a new one with every measurement.Experiment 2: Adhesive strength was evaluated using porcine hearts. Six frozen porcine hearts were defrosted and allowed to reach room temperature. A loop was made from a 1–0 surgical silk suture and was fixed to the pericardium by using a 15 mm square TC sheet with (n = 6)/without a 30 mm square PGA sheet and fibrin glue (n = 6), as in experiment 1 (Figure 1B). After stabilization by manual pressure, the suture loop was gradually elevated at a constant speed with a digital gauge (AE-1, Aikoh Engineering Co., Osaka, Japan), and the peel-off pressure (which was the maximum pressure at which the suture remained on pericardium) was recorded automatically. One porcine heart was used once for each group. The pericardium along the border of the left anterior descending artery, which was not rich in fatty tissue and not markedly curved (Figure 1B), was selected for attachment of the suture and the same site was not used again.

### Statistical analysis

Data are presented as the mean ± SD. All data were confirmed with respect to the normality of distribution using the Shapiro-Wilk test. Differences were assessed by using the paired t-test and unpaired t-test, as appropriate, with P < 0.05 being considered statistically significant. The SPSS 19 statistical software package (SPSS Inc., Chicago, IL) was used for data analysis.

## Results

**Figure 2:**
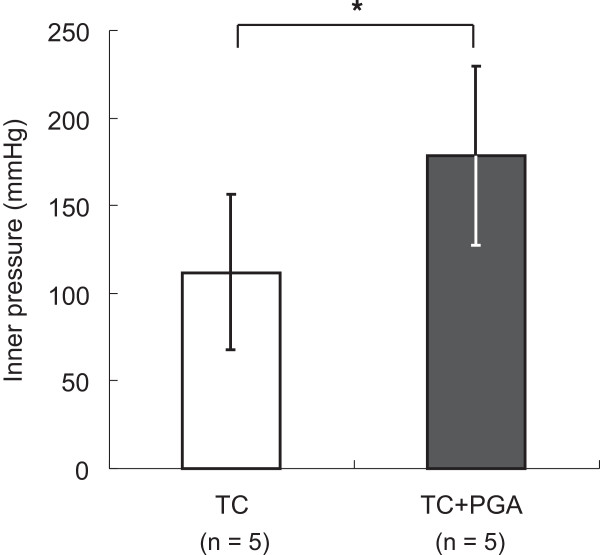
**Results of experiment 1.** *Statistically significant difference (p < 0.05), TC = A TachoComb sheet alone, TC + PGA = A PGA sheet with fibrin glue and a TachoComb sheet.

**Figure 3:**
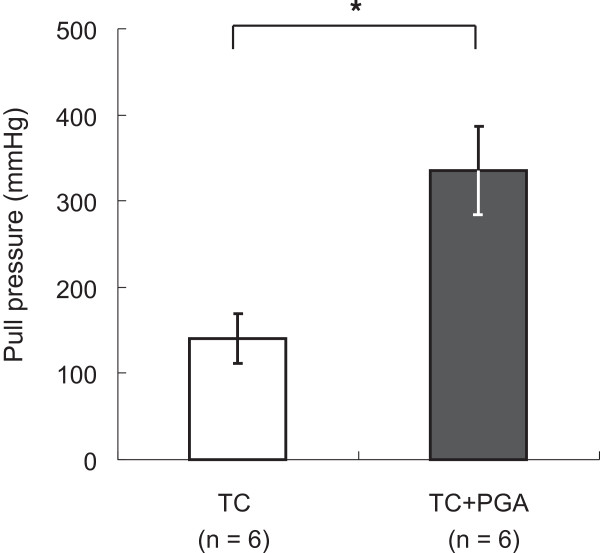
**Results of experiment 2.** *Statistically significant difference (p < 0.05), TC = A TachoComb sheet alone, TC + PGA = A PGA sheet with fibrin glue and a TachoComb sheet.

All procedures were conducted in a laboratory with a stable room temperature and humidity. In each experiment, the pressure or strain was increased gradually until the end-point was reached.Experiment 1: The PGA sheet and fibrin glue combined with a TC sheet showed a significantly higher burst pressure (178.2 ± 51.1 mmHg) when resisting the internal pressure of the airtight circuit compared with a TC sheet alone (111.6 ± 45 mmHg) (p = 0.03 unpaired t-test) (Figure 2).Experiment 2: The PGA sheet and fibrin glue combined with a TC sheet showed significantly stronger adhesion to porcine pericardium (335.2 ± 51.7 mmHg) compared with a TC sheet alone (140.2 ± 28.8 mmHg) (p = 0.0004 paired t-test) (Figure 3).

## Discussion

Haemostasis is compromised after cardiopulmonary bypass with full heparinization in patients undergoing cardiovascular surgery [3]. An increased blood transfusion volume due to bleeding during surgery or reexploration to control persistent hemorrhage is associated with a worse postoperative course and a higher mortality rate [4]. In particular, LV wall injury or LV rupture is a serious situation. For example, in the case of LV rupture associated with myocardial infarction or cardiac surgery, compromised tissue can often extend beyond the pericardial rupture site. This means that surgical repair is often difficult, and suturing can sometimes make the situation worse by enlarging the ventricular injury. In addition, if prolonged cardiopulmonary bypass and aortic cross-clamping are required to control bleeding, there is potentially a significant risk of myocardial ischemia in these compromised patients and haemostasis can be further impaired [5]. Therefore, we believe that sutureless repair under off-pump conditions is an attractive method for achieving haemostasis, while preserving coagulation and avoiding further myocardial injury.

Many authors have reported that TC (or Tachosil) is an effective alternative for controlling hemorrhage. In a case of oozing cardiac rupture, complete hemostasis could be achieved by using TC [6]. The present study showed that TC alone might be adequate for patching the myocardium, since the burst pressure was 111.6 ± 45 mmHg, and the peel-off pressure was 140.2 ± 28.8 mmHg. Even when LV rupture is not of the oozing type, a layer of TC with overlapping fascia was reported to be effective [5,7]. However, we feel that this might still not be strong enough in some cases. The surface of the left ventricle is round and uneven. Moreover the twisting contraction of the ventricular myocardium interferes with attachment of TC, making it difficult to achieve strong adhesion at sites of active hemorrhage. TachoComb sheets can be detached by the pressure of active bleeding. Even if most of a sheet remains attached to the ventricular pericardium, incomplete hemostasis due to a small gap between the sheet and pericardium caused by high-pressure bleeding is often a problem in our clinical experience. Although adding a layer of TC may be one option, it is still sometimes difficult to achieve complete haemostasis due to the limited size of TC sheets (95 × 48 mm at maximum), which means that one sheet may not have wide enough margins to be attached firmly to the ventricular surface and suppress active bleeding. The combined TC method also has a higher cost. Greater adhesive strength or extremely rapid curing of the sealant is probably necessary. Our method of using a PGA sheet and fibrin glue for reinforcement of TC can augment the adhesive strength, as demonstrated in the present study.

The PGA sheet is flexible, inexpensive and larger (150 × 150 mm) than a TC sheet, and has already been reported to be an effective haemostatic agent for lung surgery. This was the first study in which PGA sheets were applied for cardiac surgery to our knowledge. The results of the present study suggest that the PGA sheet may compensate for the weaknesses of sutureless repair using TC alone. The adhesive strength achieved with the combination of TC and PGA sheets was enough to resist the LV pressure or systolic blood pressure in the present study (335.2 ± 51.7 mmHg ≒ 446.9 ± 68.9 hPa), although it is probably impossible to achieve such high adhesive strength at a site of active bleeding with LV contraction in a critical situation. We think that the key to achieving haemostasis with TC is whether adequate compression is applied until fibrin spreads between the epicardium and the sheet and then polymerises to attach the sheet stably [8]. Adding a PGA sheet with fibrin glue augments sealing by TC alone due to covering a larger area, and PGA probably conforms during ventricular contraction without loss of flexibility. Both the PGA sheet and fibrin glue are biodegradable, so strength will decline in the chronic phase. Although there is thus a potential risk of pseudoaneurysm in the chronic phase after LV rapture, as well as the risk of pathogen transmission, to address catastrophic situations such as LV rupture and serious bleeding from other sites during cardiovascular surgery, this method might be a useful option.

## Conclusion

The present study demonstrated that adding a PGA sheet with fibrin glue to a TC sheet achieves stronger attachment than a TC sheet alone in two experimental models. Although further investigation is required, this method might be effective for haemostasis in cardiac surgery.

## Abbreviations

PGA: Polyglycolic acid; LV: Left ventricular; TC: TachoComb.

## Competing interests

The authors declare that they have no competing interests.

## Authors’ contributions

HK has made all design of this study, interpretation of data, and drafting the manuscript. IH has made all design of this study, and given final approval of this study. Both authors read and approved the final manuscript.
